# Examining the oleoylethanolamide supplement effects on glycemic status, oxidative stress, inflammation, and anti-mullerian hormone in polycystic ovary syndrome

**DOI:** 10.1186/s13048-024-01432-1

**Published:** 2024-05-22

**Authors:** Fatemeh Taghizadeh Shivyari, Hamideh Pakniat, Mohamadreza Rashidi Nooshabadi, Shaghayegh Rostami, Hossein Khadem Haghighian, Mohammad Reza Shiri-Shahsavari

**Affiliations:** 1 Department of Nutrition, Faculty of Health Qazvin, University of Medical Sciences, Qazvin, Iran; 2 Clinical Research Development Unit, Kowsar Hospital, Qazvin University of Medical Sciences, Qazvin, Iran; 3 Department of pharmacology, faculty of Medicine, Kashan University of Medical Sciences, Kashan, Iran; 4 Metabolic Diseases Research Center, Research Institute for Prevention of Non-Communicable Diseases, Qazvin University of Medical Sciences, Qazvin, Iran

**Keywords:** Oleoylethanolamide, Glycemic status, Oxidative stress, Inflammation, Anti-mullerian hormone, Polycystic ovary syndrome

## Abstract

**Objective:**

This clinical trial was designed and conducted due to the anti-inflammatory potential of Oleoylethanolamide (OEA) to examine the effect of OEA supplement on glycemic status, oxidative stress, inflammatory factors, and anti-Mullerian hormone (AMH) in women with polycystic ovary syndrome (PCOS).

**Method:**

This study was a randomized clinical trial, double-blinded, placebo-controlled that was carried out on 90 women with PCOS. Patients were divided into two groups: receiving an OEA supplement (*n* = 45) or a placebo (*n* = 45). The intervention group received 125 mg/day OEA and the placebo group received the wheat flour for 8 weeks. Demographic data were collected through questionnaires. Fasting blood sugar (FBS), insulin resistance (IR), total antioxidant capacity (TAC), malondialdehyde (MDA), C-reactive protein (CRP), tumor necrosis factor-alpha (TNF-α), and AMH were measured before and after the study.

**Results:**

Data analysis of food recall and physical activity questionnaires, showed no significant differences between the two groups (*p* > 0.05). Biochemical factors including glycemic status, MDA, inflammatory factors, and AMH decreased significantly (*p* < 0.05). TAC increased remarkably (*p* < 0.05) in comparison between the two groups, after the intervention.

**Conclusion:**

OEA supplement with anti-inflammatory characteristics could be efficient independent of diet changes and physical activity in improving disrupted biochemical factors, so both supplementation or food resources of this fatty acid could be considered as a compensatory remedy in patients with PCOS.

**Trial Registration:**

This study was retrospectively (09-01-2022) registered in the Iranian website (www.irct.ir) for registration of clinical trials (IRCT20141025019669N20).

## Introduction

Polycystic ovary syndrome (PCOS) is a prevailing endocrine disorder among women of reproductive age. It is linked to several metabolic features, including obesity, insulin resistance (IR), and hyperandrogenism [1]. It is prevalent in 4–20% of the world’s [2] and 19.5% of Iranian female population [3]. Characteristics of this syndrome include hirsutism, oligomenorrhea, compensatory hyperinsulinemia, and infertility [2]. PCOS development may relate to chronic inflammation, hyperandrogenemia, and oxidative damage, but the exact cause is still unknown [4]. This syndrome can increase the risk of cardiovascular disease (CVD) and type 2 diabetes (T2D) [2]. Common treatment strategies including medications such as metformin, anti-obesity agents, laparoscopic surgeries, or lifestyle interventions including dietary interventions, exercise, and weight control can improve outcomes [5]. However, all these methods do not provide a definitive cure for PCOS.

Hyperglycemia, a prominent complication in PCOS, can produce reactive oxygen species (ROS) that leads to oxidative stress (OS) and tissue damage [6]. Furthermore, chronic exposure to hyperglycemia can produce inflammatory factors such as tumor necrosis factor-alpha (TNF-α) and interleukin-6 (IL-6). Prolonged exposure to systemic inflammation and ROS can result in T2D, CVD [7], or PCOS [8]. Antioxidant compounds can reduce the effects of ROS by blocking inflammatory pathways and preventing cellular damage [9].

Oleoylethanolamide (OEA) is a bioactive lipid which derived from oleic acid and is a high-affinity agonist for the peroxisome proliferator-activated receptor alpha (PPAR-α). OEA showed anti-inflammatory and antioxidant properties [10]. OEA is found in small amounts (up to 2 µg/g) in cocoa powder, oatmeal, and nuts. However, some amounts of oleic acid can also convert to OEA in the human body [11]. According to current research, OEA can reduce inflammation by decreasing the mRNA expression of pro-inflammatory factors like TNF-α and IL-6 and inhibiting the nuclear factor kappa B (NF-KB) pathway [12, 13]. A study by Pouryousefi et al. found OEA supplement was effective on glycemic status in prediabetic patients [14]. Another study on female with dysmenorrhea found that OEA supplement reduced malondialdehyde (MDA), c-reactive protein (CRP), and TNF-α significantly [15].

The anti-Mullerian hormone (AMH), secreted by antral follicles, can indicate ovarian follicle reserve and function which is often elevated in PCOS [16]. Although there are limited studies on the effect of OEA on AMH, it seems that the increased level of AMH in PCOS is ameliorated in response to antioxidant and anti-inflammatory interventions. In a study by Shokrpour et al., supplementation with coenzyme Q1O as a potent antioxidant significantly decreased the AMH level in PCOS women [17]. Another study showed that vitamin D, with anti-inflammatory effects, was able to reduce AMH levels in PCOS women [18]. Since there is no specific cure for PCOS, available medicines and lifestyle modifications can only control and reduce symptoms. As mentioned, studies on the effect of OEA have shown OEA supplementation was effective in reducing inflammatory and OS factors. Therefor due to the role of inflammation in etiology of PCOS, this study aimed to evaluate the effect of OEA supplementation on glycemic status, OS, inflammatory factors, and AMH levels in women with PCOS.

## Subjects and methods

### Participants

This randomized, double-blind, placebo-controlled clinical trial involved patients with PCOS diagnosis and inclusion criteria who were selected under the supervision of a specialist physician. A total of 90 patients were included in the study, with 45 in each group.

Included patients had to meat Rotterdam criteria [19] and it requires presenting at least two out of three symptoms: hyperandrogenism, oligomenorrhea, and presents of cysts in ovaries at ultrasound scan. Also, they had to be in the age of 18 to 45 years with body mass index (BMI) in rang of 25 to 30 kg/m^2^. Patients with the following conditions are excluded from the study: Pregnancy, breastfeeding, and menopause, infectious or inflammatory disease, Cushing’s syndrome, adrenal gland tumor, hypothyroidism, increased blood prolactin, acromegaly, diabetes and cancer, hormone therapy, use of antioxidant supplements, or medicines such as contraceptives, glucocorticoids, cholesterol-lowering, and weight-reducing drugs in the last 3 months. After obtaining the consent form demographic, three-day food recall (two weekdays and one at the weekend), and International Physical Activity Questionnaire (IPAQ) [20] were completed once before and after the intervention to avoid confounding effects in diet and physical activity. Height and weight were measured and BMI were also calculated through weight in kilograms divided by the square of height in meters [21]. The Nutritionist IV program modified for Iranian food composition was used to estimate participants’ dietary intake. Data from the IPAQ were converted to metabolic equivalent-minutes/week using existing guidelines [22].

The study protocol was approved by the Ethics Committee of Qazvin University of Medical Sciences, Qazvin, Iran (ethics code: IR.QUMS.REC.1400.370) and has been documented in the Iranian Registry of Clinical Trials (http://www.irct.ir, Registration Number: IRCT20141025019669N20). All patients received and signed consent form before intervention.

### Design

Eligible patients were randomly allocated into two groups: intervention (OEA) (*n* = 45) and placebo (*n* = 45). Random numbers were used for randomized selection. Each participant received an OEA capsule (125 mg) or a placebo capsule containing 125 mg wheat flour once a day after meals for 8 weeks. The supplement capsules were similar in color, shape, and size to the placebo capsules. Since the study was double-blind, neither the patients nor the researcher and the specialist physician were aware of who received the supplement and the placebo.

The capsules for two groups were prepared by an external party and placed in identical packaging to ensure that the person administering them would be unaware of their contents. Supplements were purchased from SupplementFact Company, based in Turkey, while the placebo was created by the School of Pharmacy at Tabriz University of Medical Sciences. Patients were instructed to maintain their regular diet and physical activity routines throughout the study. The effective dosage of the OEA supplement was determined based on the research of Pouryousefi et al. [14].

To monitor their intake of OEA capsules and ensure compliance, patients were regularly contacted by phone every week. After the study, patients were required to return their supplement bottles to verify that all capsules were taken. Any patient who consumed less than 10% of the capsules was excluded from the study.

### Laboratory methods

After 10–12 h of overnight fasting, blood samples were collected from patients. Blood samples were taken two to three days after the capsules were taken. Each sample contains 10 cc of blood. The serums were separated by high-speed centrifugation and temperature of − 20 °C was used to freeze the serums. Then serum samples were stored at a − 80 °C for future laboratory evaluations. FBS concentration was measured by the enzymatic method using an Abbot ModelAclyon 300, USA auto analyzer with Pars-Azmone kit (Tehran, Iran). Insulin was measured by using a chemiluminescent immunoassay method (LIAISON analyzer (310,360) Diasorin S.P.A. Vercelli, Italy). The homeostatic model assessment for insulin resistance (HOMA-IR) (µU/ml) was calculated via formula: Fasting insulin (U/ml) × fasting blood glucose (mg/dl)/405 [14].

Serum levels of TAC was measured by a spectrophotometric method using Randox TAS (Laboratories, Crumlin, UK), by an autoanalyzer (Abbott, Model Alcyon 300, and USA). Serum MDA levels were measured by tiobarbituric acid method. Turbidimetric immunoassay was used for measuring of CRP levels (Pars Azmoon kit. Tehran, Iran). Also, enzyme-linked immunosorbent assay (ELISA) (DIAsource Co, Belgium) was used for determining serum levels of TNF-α. Serum levels of the AMH were measured by ELISA (Beckman’s kit). The normal range for serum levels of the AMH is 0.08–16 ng/ml. The mean coefficient of inter-assay and intra-assay for this method was 5.4 and 5.6%, respectively. This study was conducted as a clinical trial and blood sampling was done from people at the beginning and at the end of the study.

### Sample size calculation

In the study of Payahoo et al., TNF-α factor was associated with a significant decrease in the group receiving OEA supplement; therefore, our sample size was estimated based on this factor in the following formula [23]. The mean and standard deviation of the TNF-α before and after the supplementation was 44.19$\pm$6.3 and 20.4$\pm$4.20, so it was calculated as 40 people for each group, and 45 people were considered in each group due to the possibility of dropping out.

$$ {\text{N}} = \left[ {{{\left( {{\text{Z}}1 - \alpha /2 + {\text{Z}}1 - \beta } \right)}^2}\left( {{\text{SD}}{1^2} + {\text{SD}}{2^2}} \right)} \right]/{\Delta ^2} $$

### Statistical analysis

Statistical analyses were conducted using SPSS version 20. All data were presented as mean ± SD. The normal distribution of data was evaluated by the Kolmogorov-Smirnov test. To comparison of mean variables in each group, the statistical method of the paired t-test was used, and to compare the variables between two groups, the independent sample t-test method was used. In this research, *p* < 0.05 was considered statistically.

## Results

**Fig. 1:**
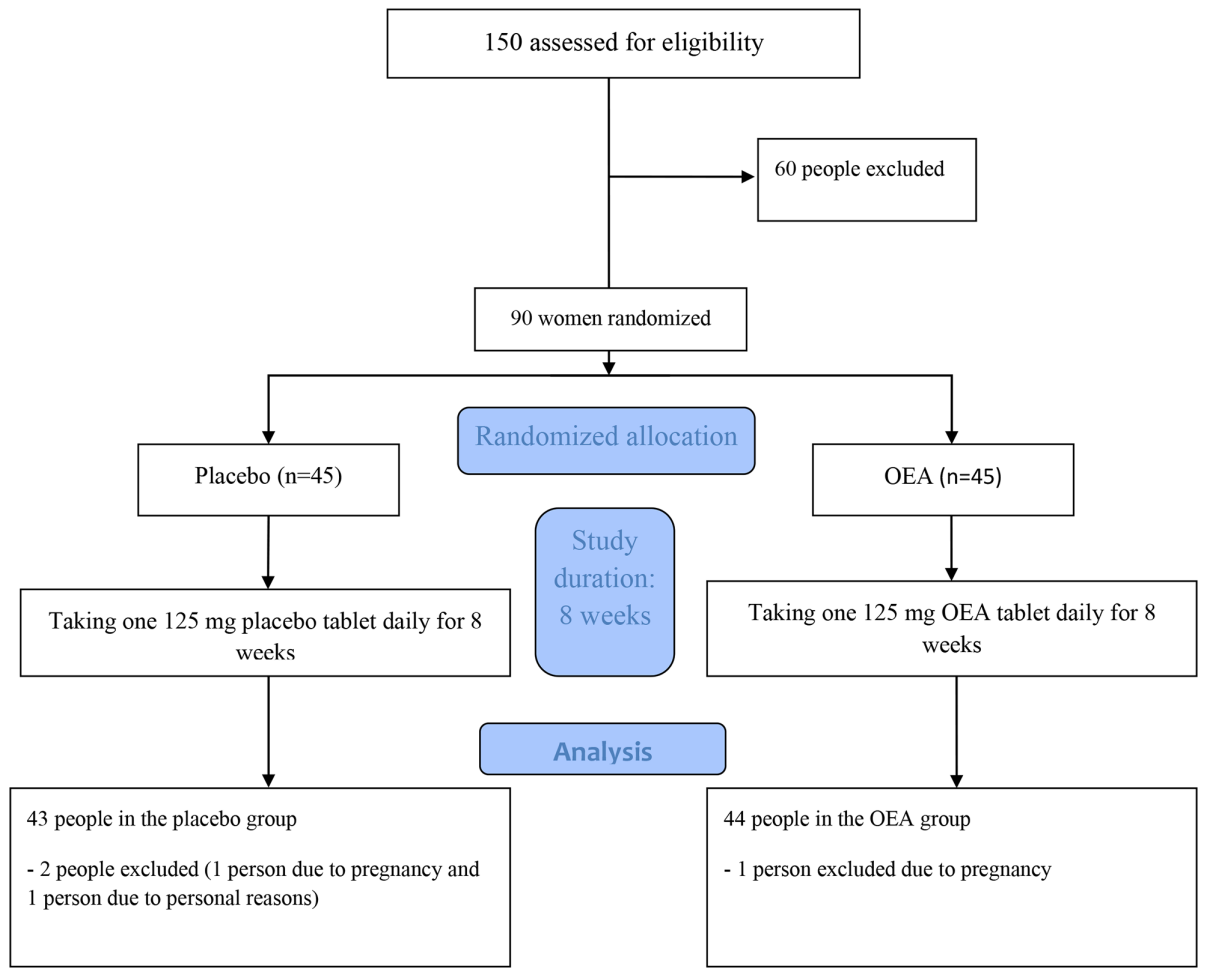
The design of the double-blind randomized trial

Ninety women with a diagnosis of PCOS enrolled in the 8-week study which was started in October 2022 and ended in February 2023. One participant from the OEA group was excluded due to pregnancy, while two from the placebo group were excluded for personal and pregnancy reasons. In the end, a total of 87 women completed the trial, with 43 in the placebo group and 44 in the OEA group (see Fig. 1). The participation rate in the study was 96.6%.

The study analyzed the data of these 87 participants, who did not differ significantly in mean age, weight, height, BMI, and physical activity levels between the two groups and they were normally distributed. Baseline characteristics are represented in Table 1.

**Table 1:** The comparison of baseline characteristics of the participants in placebo and OEA group before and after the intervention

Variable		Mean ± SD Placebo (*n* = 43)	Mean ± SD Oleoylethanolamide (*n* = 44)	P1
Age (years, Mean ± SD)		29.13 ± 5.24	27.36 ± 4.8	0.253
Height(cm)		159.26 ± 5.71	161.79 ± 4.21	0.612
Weight (kg)	Before	71.19 ± 8.23	73.5 ± 6.39	0.409
	After	70.34 ± 7.68	72 ± 8.14	0.43
	P2	0.481	0.425	
Body Mass Index (K g/m², Mean ± SD)	Before	28.06 ± 0.25	28 ± 0.36	0.501
	After	27.81 ± 0.34	27.51 ± 0.45	0.214
	P2	0.329	0.302	
Physical activity (MET minutes per week)	Before	34.19 ± 4.33	35.9 ± 5.81	0.203
	After	34.67 ± 5.74	36.1 ± 6.12	0.265
	P2	0.32	0.401	

Data are expressed as means ± SD

P1: Comparison of the mean of variables between the two groups of OEA and placebo (Independent samples t-test)

P2: Comparison of mean of variables in each group at baseline and end of study (Paired samples t-test)

The data of dietary intake is demonstrated in Table 2. Based on the collection of three-day dietary records before and after the intervention, it was discovered that there were no significant differences between the two groups or from the baseline in regards to their nutritional intake of macronutrients and some micronutrients (*p* > 0.05).

**Table 2:** The comparison of the dietary intake of the participants in placebo and OEA group before and after the intervention

Variable		Mean ± SD Placebo (*n* = 43)	Mean ± SD Oleoylethanolamide (*n* = 44)	P1
Energy(kcal)	Baseline	2179.36 ± 303.22	2257.11 ± 298.43	0.619
	End	2101.03 ± 319.09	2209.19 ± 308.11	0.683
	P2	0.656	0.693	
Protein(gr)	Baseline	88.5 ± 21.19	91.34 ± 23.5	0.203
	End	88.03 ± 21.5	91.11 ± 24.5	0.21
	P2	0.35	0.394	
Carbohydrate (gr)	Baseline	303 ± 49.5	308.12 ± 55.5	0.325
	End	302.29 ± 47.18	305.77 ± 53.09	0.38
	P2	0.45	0.401	
Fat (gr)	Baseline	69.02 ± 4.5	68.11 ± 5.13	0.367
	End	66.09 ± 6.8	69.39 ± 6.07	0.291
	P2	0.265	0.31	
Saturated fatty acids(gr)	Baseline	27.5 ± 4.21	26.15 ± 5.7	0.23
	End	25.03 ± 4.06	26.11 ± 5.1	0.207
	P2	0.19	0.311	
Monounsaturated Fatty acid (gr)	Baseline	20.08 ± 3.19	22.13 ± 3.19	0.807
	End	20.1 ± 4.19	22.17 ± 3.55	0.59
	P2	0.298	0.74	
Polyunsaturated Fatty acid (gr)	Baseline	19.63 ± 4.35	20.03 ± 4.41	0.213
	End	19.18 ± 3.13	20.16 ± 4.6	0.204
	P2	0.269	0.293	
Fiber(gr)	Baseline	9.16 ± 2.9	10.04 ± 3.33	0.136
	End	9.87 ± 2.12	10.22 ± 3.08	0.14
	P2	0.157	0.148	
Vitamin C (mg)	Baseline	69.21 ± 17.5	70.77 ± 14.32	0.415
	End	68.65 ± 19.2	70.54 ± 12.77	0.302
	P2	0.288	0.311	
Vitamin E (IU)	Baseline	11.31 ± 2.02	11.59 ± 3	0.289
	End	11.06 ± 2.31	11.24 ± 2.66	0.231
	P2	0.24	0.267	
Selenium (mcg)	Baseline	116.14 ± 29.5	117.02 ± 35.7	0.354
	End	116.02 ± 25	117.6 ± 34.61	0.329
	P2	0.39	0.402	

Data are expressed as means ± SD

P1: Comparison of the mean of variables between the two groups of OEA and placebo (Independent samples t-test)

P2: Comparison of mean of variables in each group at baseline and end of study (Paired samples t-test)

Table 3 presented the impact of OEA supplement on glycemic factors, OS, inflammatory biomarkers and AMH. At the baseline, these factors did not represent any significant differences. However, after the intervention, FBS, insulin, HOMA-IR, MDA, TNF-α, CRP, and AMH significantly decreased in the OEA group compared to the control. These factors also showed a significant reduction from the OEA group’s baseline (*p* < 0.05). Furthermore, the intervention group indicated a significant increase in the antioxidant indicator, TAC (*p* < 0.05).

**Table 3:** The comparison glycemic status, oxidative stress, inflammatory biomarkers and anti-Mullerian hormone of the participants in placebo and OEA group before and after the intervention

Variable		Mean ± SD Placebo (*n* = 43)	Mean ± SD Oleoylethanolamide (*n* = 44)	P1
FBS (mg/dL)	Baseline	105.19 ± 23.22	106.08 ± 22.54	0.577
	End	106.09 ± 24.5	92.14 ± 20.32	0.041
	P2	0.603	0.039	
Insulin (µU/ml)	Baseline	15.77 ± 3.5	15.19 ± 2.51	0.389
	End	16 ± 3.72	9.13 ± 2.22	0.0264
	P2	0.335	0.03	
HOMA-IR	Baseline	4.09 ± 0.26	3.99 ± 0.13	0.159
	End	4.19 ± 0.225	2.07 ± 0.19	0.022
	P2	0.186	0.039	
TAC (mg/dL)	Baseline	1 ± 0.022	1.06 ± 0.034	0.124
	End	1.1 ± 0.025	1.98 ± 0.061	0.023
	P2	0.109	0.017	
MDA (ng/ml)	Baseline	1.66 ± 0.039	1.59 ± 0.055	0.21
	End	1.6 ± 0.047	0.71 ± 0.026	0.03
	P2	0.209	0.028	
TNF-α (pg/ml)	Baseline	16.49 ± 4.5	16.78 ± 4.19	0.2
	End	16.22 ± 5	12.31 ± 3.36	0.041
	P2	0.208	0.036	
CRP (ng/ml)	Baseline	10.62 ± 2.8	10.14 ± 2.62	0.11
	End	10.3 ± 3.68	6.9 ± 1.59	0.029
	P2	0.19	0.031	
AMH (ng/ml)	Baseline	11.88 ± 2.19	12 ± 3.25	0.397
	End	11.94 ± 3	7.59 ± 2.13	0.048
	P2	0.35	0.041	

Data are expressed as means ± SD

P1: Comparison of the mean of variables between the two groups of OEA and placebo (Independent samples t-test)

P2: Comparison of mean of variables in each group at baseline and end of study (Paired samples t-test)

FBS: fasting blood sugar; HOMA-IR: homeostatic model assessment for insulin resistance; TAC: total antioxidant capacity; MDA: malondialdehyde; TNF-α: tumor necrosis factor-alpha; CRP: C-reactive protein; and AMH: anti-Mullerian hormone

## Discussion

Our present study found that supplementation with 125 mg/day OEA for eight weeks had a potential effect to improve glycemic, inflammatory and oxidative status in comparison to placebo (*p* < 0.05). Also, OEA caused a significant decrease in the level of AMH (*p* < 0.05).

### Effects on glycemic status

In our study, we found OEA supplement, for 8 weeks, caused a significant decrease in FBS, insulin, and HOMA-IR compared with placebo group. Our findings are consistent with Pouryousefi et al.‘s [14] findings that an eight-week OEA supplementation (125 mg/day) significantly improved glycemic status and reduced blood glucose, insulin level, and IR in pre-diabetic patients. IR can cause negative health effects in many ways. For example, it can lead to fat accumulation [24]. When adipose tissue, incredibly visceral adipose tissue, becomes insulin-resistant, insulin cannot suppress lipolysis. This situation increases circulating free fatty acids (FFAs), directly impacting liver and muscle metabolism and can aggravate IR [25]. In another study by Tutunchi et al., 125 mg OEA supplement along with a weight loss diet for 12 weeks caused a significant decrease in FBS, insulin, and HOMA-IR in patients with non-alcoholic fatty liver disease (NAFLD) [26]. Contradictory results were seen in animal studies where subcutaneous injection of 5 mg OEA/kg did not cause any significant changes in glucose levels [27].

These differences in animal and human models can depend on the dose or the way it is administered in the form of injections or supplements. The mechanism of OEA on the glycemic status can be due to the expression of the PPAR-α and G protein-coupled receptor 119 (GPR119) genes which resulted in increasing insulin sensitivity [14].

### Effects on inflammation

Our study demonstrated that CRP and TNF-α decreased significantly with 8 weeks of OEA intervention compared with placebo group. Since high levels of CRP and other inflammatory markers are commonly prevalent in PCOS, results are remarkable [28]. These findings were in consistent with Li et al.‘s study that OEA significantly reduced CRP, TNF-α, and IL-6 in NAFLD rats [29]. Akbari et al. showed that supplementation of 200 mg OEA twice a day for 14 days caused a significant reduction in CRP within and intra-group comparison [10]. Also, in Kazemi et al.‘s study, 125 mg/day OEA supplement was associated with a significant decrease in CRP and TNF-α levels in women with dysmenorrhea [15].

TNF-α can trigger hyperandrogenism [30] and CRP is along with low-grade inflammation [31]. TNF-α and CRP levels are elevated in PCOS which can lead to IR, the hallmark feature of PCOS [32, 33]. Therefore, TNF-α and CRP are used as inflammatory indices in PCOS to assess the degree of inflammation and its potential impact on the development and progression of PCOS and its associated comorbidities.

Prior findings suggested that the mechanism behind the anti-inflammatory effects of OEA may be due to the suppression of the NF-кB pathway and signal activation through PPAR-α in PCOS [34]. This finding can emphasize the anti-inflammatory effects of OEA.

### Effects on oxidation

We found a significant reduction in MDA and an increase in TAC level in OEA group after the intervention. Jin et al.‘s study demonstrated that OEA reversed lipid peroxidation and boost antioxidant capacity. OEA reduced MDA and increased serum TAC levels in the hippocampus and prefrontal cortex, resulting in antidepressant effects [33]. A study by Sabahi et al. on the effects of OEA in acute ischemic stroke showed that the intake of 300 mg/day of OEA for three days had a significant impact on increasing TAC and reducing MDA levels within the OEA group compared with the baseline [34]. Also, Kazemi et al. found significant increase in TAC and decrease in MDA levels after 60 days of 125 mg OEA compared to placebo group [15].

In contrast, the study of Payahoo et al. showed that OEA 125 mg twice a day for 8 weeks in healthy obese people did not cause significant changes in MDA and total antioxidant status (TAS). This difference may be due to obesity and having a BMI between 30 and 40 kg/m² [35].

Lipid peroxidation occurs in the plasma membrane and organelles containing lipid, creating peroxyl radicals when fatty acid side chains react with oxygen, and receive hydrogen ions from another fatty acid. Antioxidants can help pause these reactions. MDA indicates the extent of lipid peroxidation, and PCOS patients often have higher levels of lipid peroxide in their serum. TAC is a biomarker for the ability of serum to fight against free radical production [36]. Studies have shown varying levels of TAC in PCOS patients, some reporting no difference or lower levels. However, due to increased OS in PCOS, TAC levels can be reduced [37].

Activation of PPAR-alpha by OEA [10] can lead to the upregulation of antioxidant enzymes such as superoxide dismutase (SOD) and catalase, which help to neutralize ROS and prevent oxidative damage to cells. Previous studies suggested PPARα-selective ligands, such as clofibrate had protective effects by reducing ROS production and lipid peroxidation [38].

### Effects on AMH

Our study found that AMH had a significant decrease after OEA intervention compared to the placebo. Although clinical trial studies on AMH were insufficient, the anti-inflammatory and antioxidant intervention of OEA successfully reduced AMH level. Consistent with our results, in a study conducted by Shokrpour et al., the intake of coenzyme Q10 supplement led to a significant decrease in the AMH levels in women with PCOS [17]. Another study has demonstrated that vitamin D can also effectively lower the AMH levels in PCOS [18]. These results emphasize the importance of anti-inflammatory and antioxidant interventions in reducing AMH in women with PCOS.

Other studies indicated the role of OEA on fertility of men. For example, in a study by Ambrosini et al. they investigated the protective effect of OEA on sperm. The results of that study showed that OEA protected sperm against ROS. The mechanism of this action is assumed to be through the binding of OEA to serum albumin. Albumin plays an important role in sperm quality and due to its antioxidant properties, it improves sperm quality. On the other hand, OEA can bind to serum albumin with high affinity and protect serum albumin from oxidation caused by ROS.

AMH level indicates ovary supply and mostly increases in PCOS. The rise in AMH level is from accumulation of follicles which is responsible for 2 to 4-fold increase in AMH in PCOS. AMH is in connection with other pituitary hormones like follicle-stimulating hormone (FSH) that has a stimulating results on AMH production [39].

### Effects on weight loss

Our participants did not show any significant weight loss after OEA intervention. Many women with PCOS struggle with being overweight or obese, with a rate of 60% experiencing such issues. Unfortunately, obesity can worsen PCOS symptoms, as the BMI increases by 1 point, the likelihood of developing PCOS also increases by 9% [40]. According to the study conducted by Laleh et al., when 56 obese individuals (with a BMI between 30 and 40 kg/m^2^) were given two daily supplement of 125 mg OEA for 60 days, their weight, appetite and BMI were remarkably reduced by the end of the study [41]. The lack of significant weight loss could be due to the difference in OEA dosage.

However, our study had certain limitations. The duration of the intervention was relatively short, and measuring additional biochemical factors could provide a better understanding of the effects of OEA on PCOS. Additionally, exploring the effects of lower or higher doses of OEA could help determine the optimal dosage. In addition, because this study was conducted for the first time on the effect of OEA on PCOS, there were no previous studies in this field to compare the results with. Additionally, there was a lack of research on the effects of OEA on other women’s diseases.

To the best of our knowledge, this study is the first to investigate the effects of OEA on PCOS. We had a high participation rate, and the randomization of participants into two groups ensured that there were no significant differences in baseline characteristics. Our study found that a dose of 125 mg/day of OEA over an 8-week period, independent of diet and physical activity, led to significant improvements in reducing disrupted biochemical markers.

We recommend that future studies with more extended follow-up periods assess apparent symptoms such as hirsutism and regulation of periods, as well as explore the molecular mechanism of OEA on ovaries. The role of overweight or obesity in PCOS, food intake and anthropometric indices should also be considered in future studies. The present study had the strenghth and waek points. According to the search in the database, this study was conducted for the first time, which could potentially lead to new treatment options for PCOS. This study can serve as a valuable starting point to encourage further research in this field. All stages of this study were designed and implemented with the help of reliable sources and valid tools. Double blinding and randomization were also considered as the strengthsThe limitation of studies exploring the impact of OEA on women’s health rendered the task of comparing results a challenging one. The insufficiency of research in this area underscores the need for further investigation to gain a comprehensive understanding of the effects of OEA on women.

## Conclusion

In the present inquiry, our intention was to evaluate the effect of OEA on several factors in women with PCOS, including glycemic status, OS, inflammatory factors, and AMH. The results showed significant improvement in biochemical characteristics. The findings support the importance of antioxidant and anti-inflammatory interventions in managing PCOS. To the best of our knowledge, this is the first study to explore the effects of OEA on PCOS in women, and hence, further research is necessary to confirm these findings through more interventions.

## Data Availability

We make sure that all data and materials support our published claims and comply with field standards.

## Abbreviations

AMH: Anti-mullerian hormone
BMI: Body Mass Index
CRP: C-reactive protein
CVD: cardiovascular disease
ELISA: Enzyme-Linked Immunosorbent Assay
FBS: Fasting Blood Glucose
FFAs: Free fatty acids
IPAQ: International Physical Activity Questionnaire
IL-6: Interleukin-6
IR: insulin resistance
MDA: Malondialdehyde
NF-кB: Nuclear factor-kappa-B
NAFLD: Non-alcoholic fatty liver disease
OEA: Oleoylethanolamide
OS: Oxidative stress
PCOS: Polycystic ovary syndrome
ROS: Reactive Oxygen Species
TAC: Total antioxidant capacity
TNF-α: Tumor Necrosis Factor Alpha
T2D: Type 2 diabetes
